# The SHEDS-Wood Model: Incorporation of Observational Data to Estimate Exposure to Arsenic for Children Playing on CCA-Treated Wood Structures

**DOI:** 10.1289/ehp.9741

**Published:** 2007-02-21

**Authors:** Leila M. Barraj, Joyce S. Tsuji, Carolyn G. Scrafford

**Affiliations:** 1 Center for Chemical Regulation and Food Safety, Exponent, Inc., Washington, DC, USA; 2 Center for Toxicology and Mechanistic Biology, Exponent, Inc., Bellevue, Washington, USA

**Keywords:** arsenic, CCA, children’s exposure, probabilistic exposure modeling, observational studies, SHEDS-Wood

## Abstract

**Background:**

Lumber treated with chromated copper arsenate (CCA) compounds has been used in residential outdoor wood structures and public playgrounds. The U.S. Environmental Protection Agency (EPA) has conducted a probabilistic assessment of children’s exposure to arsenic using the Stochastic Human Exposure and Dose Simulation model for the wood preservative scenario (SHEDS-Wood). The assessment relied on data derived from an experimental study conducted using adult volunteers and designed to result in maximum hand and wipe loadings to estimate the residue–skin transfer efficiency. Recent analyses of arsenic hand-loading data generated by studies of children actively involved in playing on CCA-treated structures indicate that the transfer efficiency coefficient and hand-loading estimates derived from the experimental study significantly overestimate the amount that occurs during actual play.

**Objectives:**

Our goal was to assess the feasibility of using child hand-loading data in the SHEDS-Wood model and their impact on exposure estimates.

**Methods:**

We used data generated by the larger of the studies of children in SHEDS-Wood, instead of the distributions used by U.S. EPA. We compared our estimates of the lifetime average daily dose (LADD) and average daily dose (ADD) with those derived by the U.S. EPA.

**Results:**

Our analysis indicates that data from observational studies of children can be used in SHEDS-Wood. Our estimates of the mean (and 95th percentile) LADD and ADD were 27% (10%) and 29% (15%) of the estimates derived by U.S. EPA.

**Conclusion:**

We recommend that the SHEDS-Woods model use data from studies of children actively playing on playsets to more accurately estimate children’s actual exposures to CCA.

Chromated copper arsenates (CCA) are chemical preservatives used to pressure-treat wood to protect it from rotting due to moisture, insects, and microbial agents. CCA-treated wood has been used widely in outdoor residential structures (e.g., decks, porches, playsets, landscaping) and public playgrounds for the last three decades. In 2002, the U.S. Environmental Protection Agency (EPA) announced a voluntary decision by the pressure-treated wood industry to phase out the use of CCA in products destined for consumer markets; however, existing outdoor wood structures made with CCA-treated wood remain in service. As a part of the reregistration review of CCA-treated wood by the U.S. EPA under the Federal Insecticide, Fungicide, and Rodenticide Act (FIFRA), the U.S. EPA Office of Research and Development (ORD) National Exposure Research Laboratory (NERL) developed the Stochastic Human Exposure and Dose Simulation model for assessing residential and playground exposures to wood preservatives (SHEDS-Wood) and applied it to assess children’s exposure to arsenic from CCA-treated structures (U.S. EPA 2005; Xue et al. 2006; Zartarian et al. 2006).

The SHEDS-Wood model is a probabilistic model designed to simulate aggregate exposures and doses for population cohorts and multimedia chemicals, by using data from time–location–activity diaries compiled in U.S. EPA’s Consolidated Human Activity Database (CHAD) (McCurdy et al. 2000). The model input parameters are grouped in four categories: *a*) activity factors (including parameters such as the average number of days that a child plays on or near playsets/decks, fraction of time a child is actually on the play-set/deck), *b*) exposure factors (including frequency of hand-to-mouth activity, washing events, dermal loading, residue skin transfer efficiency), *c*) dose factors (including dermal and gastrointestinal absorption fractions), and *d*) environmental media parameters (including soil and residue concentrations). The values assigned to the input parameters in the U.S. EPA’s and Zartarian et al.’s assessments were from data generated by *a*) experimental studies (e.g., for the dermal loading variable and the residue skin transfer efficiency), *b*) observational studies (e.g., for frequency of hand-to-mouth activity and the fraction of hand mouthed), or *c*) expert judgment (e.g., for the number of days on playgrounds and the fraction of time spent on playsets).

Among these parameters, the model is most sensitive to the amount of arsenic that accumulates on a child’s hand. Sensitivity and uncertainty assessments conducted by the U.S. EPA (Xue et al. 2006) indicated that the residue skin transfer efficiency and the wood surface arsenic residues—the parameters used to estimate maximum loadings in the U.S. EPA’s (2005) and Zartarian et al.’s (2006) analyses—are the two most important variables influencing the estimation of absorbed doses. The U.S. EPA’s SHEDS-Wood model, as well as other assessments of childhood arsenic exposure from treated wood [e.g., Consumer Product Safety Commission (CPSC) 1990; Hemond and Solo-Gabriele 2004], have relied on exposure assessments conducted using assumptions and modeling, rather than using actual levels of arsenic on children’s hands after playing on structures made with CCA-treated wood. The report of the FIFRA Scientific Advisory Panel (SAP) Meeting (U.S. EPA 2001), held 23–25 October 2001 to review the U.S. EPA’s preliminary assessment of exposures to CCA-treated wood, recommended “that the EPA conduct direct hand-loading measurements in samples of children (preferably) or adults (if human subjects concerns intervene). The best empirical data may actually be collected through sampling of children who are actively involved in playing on CCA treated structures.”

Two recent observational studies improve the assessment of exposure by directly measuring the level of arsenic on children’s hands after contact with CCA-treated wood. The first study by Kwon et al. (2004), with follow-up by Wang et al. (2005), included 130 children (66 playing on playgrounds with CCA-treated playsets and 64 playing on playgrounds with playsets not constructed with CCA-treated wood); the second (Shalat et al. 2006) included 11 children playing on residential playsets (seven children on wood structures that were at least partially CCA-treated and four on non-CCA-treated structures). Zartarian et al. (2006) reviewed the hand-loading estimates derived from the Kwon et al. study and noted that these data suggest “that experimental methods used to measure hand loadings may overestimate the amount that occurs during actual play.” We obtained the raw data from the authors of the first study (Kwon et al. 2004) and conducted an assessment to determine whether these data can be used in SHEDS-Wood, and if so, what impact they will have on the estimates of the model’s predicted lifetime average daily dose (LADD) and intermediate-term average daily dose (ADD).

## Materials and Methods

### The SHEDS-Wood model

The SHEDS-Wood model estimates exposure to and dose of arsenic using age- and sex-representative time–location–activity diaries for 1- to 6-year-old children extracted from the CHAD database. Each of the diaries used includes different macro-activities of children over the course of a day. The macro-activities last from a few minutes to an hour, during which time potential contact with CCA-treated playsets or decks may occur. Because the macro-activities reported in CHAD are not sufficiently detailed to indicate exactly whether and when contact with CCA-treated wood occurs, SHEDS-Wood models the contact with CCA-treated wood probabilistically in a subset of the macro-activities that take place in what it defines as “suitable locations.” Pathway-specific exposure and dose time profiles are then generated from the sequence of contact events.

Contact or encounter can occur only at places where the chemical is present. Once such contact occurs, the chemical remains present on or in an individual until it is removed or excreted. The removal processes in SHEDS-Wood involve dermal absorption, oral ingestion (through hand-to-mouth activity), washing, and bathing, and are assumed to occur sequentially in this order. In addition, the model assumes that the gastrointestinal (GI) tract is voided once per day at 0600, at which time the GI tract loading is reset to zero. SHEDS-Wood includes an adjustment to limit the net transfer of chemical from the wood to the skin, at a point at which a maximum hand load of arsenic has been reached after multiple contacts with CCA-treated wood surfaces. The data used to estimate the distribution of maximum hand loadings were generated by an experimental study in which adult volunteers rubbed CCA-treated wood blocks with their hands (for 20 passes), and polyester wipes were pulled back and forth across the blocks (10 passes, followed by a 90° rotation, and an additional 10 passes) [American Chemistry Council (ACC) 2003]. A distribution representing the residue–skin transfer efficiency parameter was derived as the ratio of the hand-rinse results to the wood-block residue (cloth wipe) results. SHEDS-Wood models the maximum hand loading as the product of the wood surface residue concentration times the transfer efficiency (“default” option) or as a user-specified distribution. The U.S. EPA (2005) used the default SHEDS-Wood model option in its “base” analysis, and used the hand wipe data generated by the ACC experimental study in a “special analysis” to assess the impact of using “dislodgeable residues directly rather than total residues multiplied by transfer efficiency.”

### The Kwon et al. study

The Kwon et al. (2004) study was conducted in Edmonton, Alberta, Canada, and was supported in part by the Natural Sciences and Engineering Research Council, the City of Edmonton, and Environment Canada; study protocols were approved by the University of Alberta Health Research Ethics Board. Sixteen of the 316 playgrounds owned and operated by the City of Edmonton were selected for the study. The playgrounds selected were representative of other playgrounds in the City of Edmonton with respect to age, manufacturers, and geographic location. Eight of the playgrounds contained CCA-treated wood structures, and the other eight did not. A total of 130 children (66 playing on playgrounds with CCA-treated playsets and 64 playing on playgrounds with playsets not constructed with CCA-treated wood) participated in the study. On average, seven to nine children participated at each playground. Written informed parental consent was obtained; children for whom parental consent was not obtained were excluded from the study. The time of arrival at the playground, the length of play, and the ages of the participating children were recorded. After the children finished playing, their hands were rinsed with de-ionized water, and the hand-washing samples were filtered and the filtrate was analyzed. The data summaries were published by Kwon et al. (2004). In response to comments by Kissel (2005), the arsenic levels in the insoluble residue collected on the filter were analyzed, and the results were published by Wang et al. (2005) in rejoinder. From here on, we will use “Kwon et al.” to refer to the data from the Kwon et al. and the Wang et al. studies.

### Hand-loading data

The raw data on total arsenic (soluble arsenic + insoluble arsenic on hands) in hand washings from each of the 130 children were obtained from the study authors (Lee XC, personal communication). The total arsenic collected in the hand-washing water (insoluble arsenic on the filter plus water-soluble arsenic in the filtrate) was 934 ± 940 ng for the children playing on the CCA-treated playgrounds and 265 ± 311 ng for the children playing on the non-CCA-treated playgrounds. We used the total arsenic data available for the children playing on CCA-treated playgrounds and did not adjust these data to reflect the background arsenic levels detected on the hands of children playing on non-CCA-treated playgrounds.

Of the 66 children playing on the eight CCA-treated decks, 53 were 1–6 years of age, the age range considered in the assessments by the U.S. EPA (2005) and Zartarian et al. (2006). The remaining 13 children (one child 8 months of age, and 12 children ≥ 7 years of age) were outside the age range considered in U.S. EPA’s assessment; thus, their data were not included in our analysis. Further, data were also excluded from one 2-year-old child whose soluble arsenic value was considerably lower than the levels measured for the other children and for whom no insoluble arsenic level was reported. In this analysis, we used the total arsenic data available for the remaining 52 children 1– 6 years of age playing on CCA-treated playgrounds.

### Data analysis

The Kwon et al. hand-loading data are in total arsenic mass (for two hands), whereas the maximum hand-loading parameter in the SHEDS-Wood model is expressed as arsenic mass per square centimeter of skin. Hence, hand-loading levels in the Kwon et al. study were transformed from total arsenic loadings for two hands to micrograms arsenic per square centimeter of skin for use in the SHEDS-Wood model, using formulas and assumptions similar to those used by the model. Specifically, it was assumed that 50.5% of the time spent on the playground was actually spent on the play structure, as opposed to “near” the play structure (F_playset_), and that 74% of the hand skin surface area contacts wood surface residues per 20 min (F_contact,res,j_) (these percentages correspond to the averages of the distributions used in SHEDS-Wood for these parameters). Estimates of the hand (HSA) and total body surface areas (TBSA) were derived using formulas similar to those used in SHEDS-Wood (Table 1). Thus, arsenic hand loading per square centimeter were derived as:


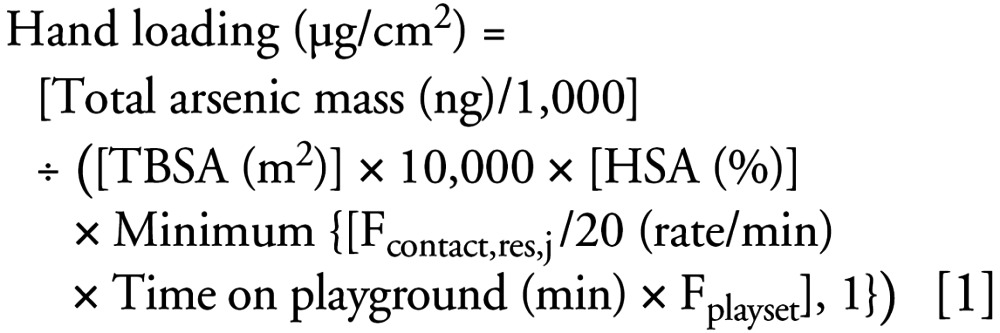


Because the assumption that 74% of the hand skin surface area contacts wood surface residues per 20 min implies that 100% of the hand would contact residues after about 30 min, the above formula caps this parameter at 1 for children spending > 1 hr on the playground. We used linear regression analysis to assess the association between arsenic hand loadings and time spent on the playgrounds and used SHEDS-Wood to estimate LADD and intermediate-term (90-day) ADD for children playing on CCA-treated wood structures.

## Results

### Arsenic hand washing data and association with time on playground

Table 2 summarizes the distribution of total arsenic in hand-washing data from the Kwon et al. study (nanograms) and the transformed distribution (micrograms per square centimeter) and compares these distributions with the hand-loading distribution used by U.S. EPA in SHEDS-Wood (U.S. EPA 2005). The geometric mean and 95th percentile estimate of the hand-loading distribution derived from the Kwon et al. study (0.0016 μg/cm^2^ and 0.0091 μg/cm^2^) are more than an order of magnitude lower than those of the distribution used by U.S. EPA in SHEDS-Wood (0.0334 μg/cm^2^ and 0.1061 μg/cm^2^).

**Figure 1:**
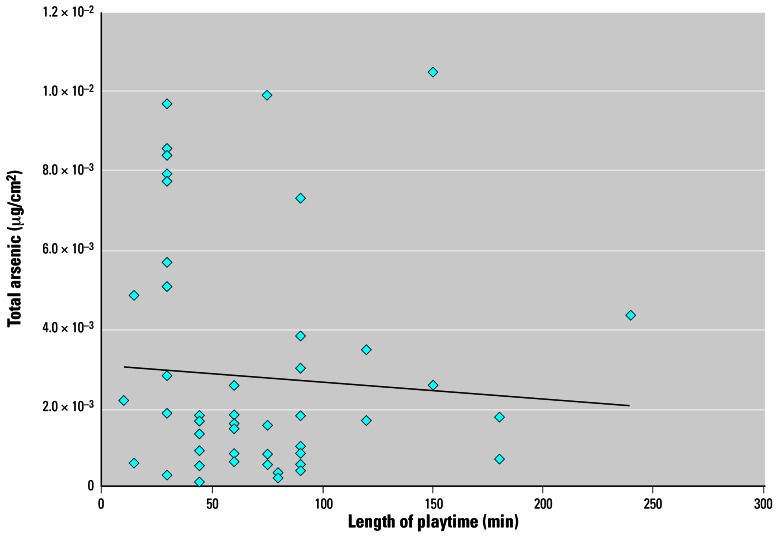
Total arsenic in hand washings and time on structure (children 1–6 years of age). Linear regression line: *Y* [total arsenic (μg/cm^2^)] = –4 × 10^−6^; *X* [length of playtime (min)] + 0.0031 (*R*^2^ = 0.0048).

The regression analysis indicated that total arsenic levels showed little correlation (*R*^2^ = 0.0048) with the length of play time (Figure 1), suggesting that hand loading achieves a steady-state level after a short period of play. This result is consistent with the U.S. EPA’s conclusion that saturation was achieved in the ACC experimental adult study (U.S. EPA 2005). The distribution of hand loadings derived from this study can thus be used in SHEDS-Wood to represent maximum hand loading of arsenic in lieu of the distribution used by U.S. EPA and the distribution derived from the experimental data in adults.

### LADD and ADD estimates

We derived estimates of the LADD and intermediate-term (90-day) ADD for the upper-bound (“warm climate”) scenario in SHEDS-Wood using the same values and distributions as those used by U.S. EPA’s (2005) assessment for all input parameters, except for the maximum dermal loading parameter, for which we used the maximum value of the arsenic hand loadings (expressed in micrograms per square centimeter) derived from the Kwon et al. data. Table 3 summarizes the values and distributions used for the input parameters in SHEDS-Wood.

Tables 4 and 5 summarize estimates of the LADD and the intermediate-term ADD derived using the maximum value for hand loading based on the Kwon et al. study and compare these estimates with those derived by the U.S. EPA (2005) for children playing on CCA-treated decks and playsets. The mean LADD estimates derived by setting the maximum dermal loading parameter at values derived from the Kwon et al. study were 27% of the estimates derived by the U.S. EPA. Larger differences were observed between the U.S. EPA’s estimates of the upper-percentile exposure distributions and the estimates derived using the Kwon et al. study data. Specifically, the 90th and 99th percentile estimates derived by setting the maximum dermal loading parameter at values derived from the Kwon et al. study were 14% and 10% of the estimates derived by the U.S. EPA. Similarly, mean, 90th, and 95th percentile ADD estimates derived by setting the maximum dermal loading parameter at values derived from the Kwon et al. study were 29%, 19%, and 15%, respectively, of the estimates derived by the U.S. EPA (2005).

## Discussion

The data used by the U.S. EPA (2005) and Zartarian et al. (2006) in SHEDS-Wood to represent transfer efficiency and maximum dermal loadings were derived from an experimental study conducted on adults that was designed to achieve the maximum hand and wipe loading of dislodgeable arsenic from CCA-treated wood. Particularly, the study used wetted hands, weights, a large number of passes, and no allowance for brush-off. Such a study does not reproduce typical children’s exposure on playsets. The FIFRA SAP Meeting (U.S. EPA 2001) had recommended that direct hand-loading measurements for children who are “actively involved in playing on CCA treated structures” be obtained and used. The Kwon et al. study (2004; Wang et al. 2005) provides such data and indicates that the experimental data used by the U.S. EPA (2005) and Zartarian et al. (2006) overestimate the arsenic residue–skin transfer efficiency factor and the arsenic hand loadings that occur during actual play. The geometric mean and maximum hand-loading levels from the combined soluble and insoluble arsenic were 0.3 μg and 4.7 μg, respectively. If we assume, conservatively, that only the palms of the children’s hands contacted the residue, the adjusted geometric mean and maximum of the hand loadings become 0.004 μg/cm^2^ and 0.036 μg/cm^2^, respectively, much lower than the estimates derived from the lognormal distribution used by the U.S. EPA (2005) and Zartarian et al. (2006) (geometric mean: 0.033 μg/cm^2^; 99th percentile: 0.272 μg/cm^2^). Using assumptions similar to those used in SHEDS-Wood for body and hand surface areas, time spent on structure, and fraction of hand skin surface area contacting residues, to calculate the hand loadings per square centimeter results in even lower levels (Table 2). Furthermore, the hand-loading levels observed in the Kwon et al. study are consistent with those observed in a smaller but similar study conducted by Shalat et al. (2006).

Zartarian et al. (2006) conclude that the U.S. EPA-estimated ADD is equivalent to what the ingested dose would be for the Kwon et al. study (Kwon et al. 2004; Wang et al. 2005). However, their conclusion is based on the assumption that all the arsenic on the children’s hands is ingested and absorbed completely. This conservative assumption is contradicted by the inputs used in SHEDS-Wood by the U.S. EPA (2005) and Zartarian et al. (2006) to model exposure—namely, the fraction of dermal absorption, the fraction of gastrointestinal absorption, and the fraction of hand surface area mouthed by mouthing-event parameters.

The mean of the background arsenic levels on children’s hands, derived from hand washings of children playing on non-CCA-treated playgrounds, was 26% of the mean levels detected on the hands of children playing on CCA-treated playgrounds. In our analysis, we did not adjust the total arsenic collected in the hand-washing water for the children playing in CCA-treated playgrounds to reflect the background levels, and hence, may have overestimated arsenic exposure that may arise from playing in CCA-treated playgrounds.

As with all observational studies, the Kwon et al. study (Kwon et al. 2004; Wang et al. 2005) has certain limitations. However, those limitations do not prevent use of the data to inform the exposure assessment. Potential limitations of the Kwon et al. study include the relatively small number of children, the fact that the study recorded time spent on the playgrounds, not time spent on the playsets, and the fact that the study did not include surface wipe analyses of the structures; hence, it is not possible to confirm that dislodgeable arsenic levels from the play structures in the Kwon et al. study are not lower than those measured in other studies. We address each of these concerns below.

### Sample size

Using a one-sided tolerance interval approach, similar to that described in Hahn and Meeker (1991) and proposed by the U.S. EPA (1991), for estimating the minimum sample necessary when the quantity to be estimated from a survey is a percentile of a distribution, it can be shown that the number of children playing on playgrounds with CCA-treated playsets (*n* = 66) in the Kwon et al. study is large enough to allow estimation of the 95th percentile of the distribution of hand loadings with at least 95% confidence. This sample size is larger than what is typically available for observational studies of children, such as those used by the SHEDS-Wood model. For instance, SHEDS-Wood relies on data from Kissel et al. (1998) to support the fraction of the unclothed body and hand skin that contacts residues. The Kissel et al. study used a fluorescent tracer to study the soil loading on 12 children wearing short pants and short sleeves who played in soil for 20 min. The study not only included a much smaller number of children (*n* = 12) than did the Kwon et al. study, but it also refers to exposures that are of limited relevance to the exposure scenario in SHEDS-Wood, because the surface area of the hand that contacts hard surfaces such as playsets and decks is expected to be much smaller than the surface area that contacts soil. Similarly, SHEDS-Wood uses data from Leckie et al. (2000) to support the fraction of hand surface area mouthed per mouthing event and the frequency of hand-to-mouth activity. The sample size for this study consisted of 20 children, much smaller than the number of children included in Kwon et al.

### Time on playsets was not recorded

Although it is not possible to confirm that the children in the Kwon et al. study actually were on the playsets, significant differences were observed between hand loadings of children playing in playgrounds on CCA-treated play-sets and of children playing in playgrounds with no CCA-treated playsets. Thus, even if some of the children in the Kwon et al. study did not play on the playsets, the data indicate that many of the children did play on the play-set, particularly the children with the higher hand loadings. In our assessment, we used the maximum loading from the Kwon et al. study. The SHEDS-Wood model includes an adjustment to represent the probability that not all outdoor activities in what it considers potential playset locations do result in actual contact. Specifically, the SHEDS-Wood model includes such an adjustment, because a fraction of the activities recorded in CHAD at the locations considered by the model to be suitable for playset contact are not actually playset activities. We did not make this adjustment when the hand-loading levels were transformed from total arsenic loadings for two hands to micrograms arsenic per square centimeter of skin for use in the SHEDS-Wood model, because the children in the Kwon et al. study were on actual playgrounds, thus engaging in activities likely to result in contact with the treated structures.

### No surface wipe analyses

Although the Kwon et al. study did not include wipe analyses, data from other studies that did measure dislodgeable arsenic on wood structures from various geographic areas show consistent levels [ACC 2003 (samples collected from structures in Pennsylvania, Florida, and Georgia); CPSC 2003 (samples collected from structures in the Washington, DC, metropolitan area); Shalat et al. 2006 (samples collected from structures in Florida); Ursitti et al. 2004 (samples collected from structures in Toronto, Canada)]. Hence, we have no reason to believe that the dislodgeable arsenic levels from the structures in the Kwon et al. study would be different from those reported in these studies.

Kissel (2005) point out that a potential limitation of the Kwon et al. study is that the hand-loading levels derived from the study may not reflect the amount already ingested. However, even if we adjust the levels derived from Kwon et al. using the mean of the distribution of hand-to-mouth dermal transfer fraction assumed in SHEDS-wood (0.78)—thus in fact assuming that the children in the Kwon et al. study licked the entire area of their hand that had contacted the arsenic residues—the levels used by the U.S. EPA (2005) would still be 50–80% higher than these adjusted levels. Hence, the potential amount removed by mouthing is unlikely to account for the difference we observed between our exposure estimates and those derived by the U.S. EPA (2005), particularly because we have conservatively used the maximum value derived from the Kwon et al. data in our analysis.

The follow-up sensitivity and uncertainty analyses conducted by the U.S. EPA and Xue et al. (2006) indicate that residue–skin transfer efficiency and wood surface arsenic residues—the parameters used to estimate maximum loadings in the U.S. EPA (2005) and Zartarian et al. (2006) analyses—are the two most important variables influencing the estimation of absorbed doses. Therefore, hand-loading data collected from children during active play, such as those collected by Kwon et al. (2004), Wang et al. (2005), and Shalat et al. (2006), should be used to improve the accuracy of the dose and exposure estimates derived by the U.S. EPA using the SHEDS-Wood model.

## Conclusion

We obtained the total arsenic hand-loading data from the Kwon et al. study and analyzed them to assess whether these data can be used in SHEDS-Wood to represent the maximum hand-loading parameter. Our analysis indicates that the measured hand loadings were not associated with the amount of time spent on the playground, suggesting that hand loading achieves a maximum level after a short period of play, and thus that these data can be used in the SHEDS-Wood model in lieu of the values used by the U.S. EPA (2005) and Zartarian et al. (2006) that depend on the experimental study.

Using data collected through sampling of actual children playing on CCA-treated structures, such as the data from the Kwon et al. study, in SHEDS-Wood results in mean LADD and ADD estimates that are 27% and 29%, respectively, of the estimates derived by the U.S. EPA. Larger differences are observed at the upper percentiles, where our estimates are 14% and 19% (90th percentile) and 10% and 15%, respectively, of the estimates derived by the U.S. EPA (2005). Based on our analysis of child hand-loading data from observational studies of children, the U.S. EPA’s current assessment overestimates children’s potential exposure to dislodgeable arsenic from CCA-treated wood by up to 10-fold (U.S. EPA 2005). We recommend that the U.S. EPA use the data from this observational study in SHEDS-Wood to more accurately estimate children’s actual exposure to dislodgeable arsenic. Our recommendation is consistent with the FIFRA SAP recommendation that the best empirical data for hand loading are those collected from children who are actively involved in playing on treated wood structures.

## Figures and Tables

**Table 1:** Formulas used to estimate the hand and body surface areas of the children in the Kwon et al. study.

Parameter	Sex and age (years)	Formula or value used
Body weight and height	Males: ≤ 6	Height = 73.61 + 0.6068 × age (in months) Body weight = exp (0.7487 + 0.0202 × height)
	Females: ≤ 6	Height = 72.05 + 0.6231 × age (in months) Body weight = exp (0.7077 + 0.0205 × height)
TBSA	Males and females: ≤ 5	Surface area (m^2^) = 0.02667 × height^0.38217^ × weight^0.53937^
	Males and females: > 5	Surface area (m^2^) = 0.0305 × height^0.35129^ × weight^0.54375^
HSA (% of total surface area)	Males and females: ≤ 4	6
	Males and females: 5	5.5^a^
	Males and females: > 5	5

a The algorithm used by SHEDS-Wood assumes 5% for persons > 4 years of age; however, using 5% resulted in a smaller average hand surface area for children 5 years of age than for those 4 years of age, so we used the average value of 5.5% for this age group.

**Table 2:** Comparison of total arsenic in hand washings for children in Kwon et al. to the distribution used by U.S. EPA in SHEDS-Wood.

	Total arsenic in hand washings^a^		
Percentile	As measured (ng)	Transformed^b^ (μg/cm2)	Distribution used by U.S. EPA (μg/cm^2^)
25th	278	0.0008	0.0182
50th	566	0.0016	0.0334
75th	1,216	0.0036	0.0614
90th	1,996	0.0079	0.0714
95th	2,576	0.0091	0.1061
99th	4,427	0.0102	0.1472
Maximum	4,743	0.0105	—
Geometric mean	321	0.0016	0.0334
Geometric SD	2.7	2.9138	2.4624

a Estimates derived for children 1–6 years of age only.

b Estimates derived using assumptions similar to those used in SHEDS-Wood.

**Table 3:** Input parameters in the SHEDS-Wood Model.

Variable	Unit	Distribution
Fraction children with CCA-treated home playset		Point (0.08)
Average fraction residential outdoor time a child plays on/around CCA-treated residential playset		Beta (1.1, 0.36)
Average no. of days/year a child plays on/around residential CCA-treated playset	Days/year	Point (126)
Average fraction nonresidential outdoor time a child plays on/around CCA-treated public playset		Beta (1.1, 0.36)
Average no. of days/year a child plays on/around treated CCA-treated public playset	Days/year	Point (126)
Fraction time a child on/around treated playset is on playset itself vs. on ground near playset		Beta (12.35, 12.12)
Fraction children who have a CCA-treated residential deck		Point (0.5)
Average fraction residential outdoor time a child plays on/around CCA-treated residential deck		Beta (1.1, 0.36)
Average no. of days/year a child plays on/around CCA-treated residential deck	Days/year	Point (126)
Fraction time a child on/around CCA-treated home deck is on the deck vs. on the ground near the deck		Beta (39.2, 4.3)
Soil concentrations near CCA-treated playset	mg/kg	Lognormal (29.96979, 1.643408)
Wood surface residues on CCA-treated playset	μg/cm^2^	Lognormal (0.228262, 2.242383)
Soil concentrations near CCA-treated deck	mg/kg	Weibull (1.056996, 41.89868)
Wood surface residues on CCA-treated deck	μg/cm^2^	Lognormal (0.228262, 2.242383)
Residue–skin transfer efficiency		Lognormal (0.143203, 2.3307)
Fraction of total body (non-hand) skin surface area that is unclothed		Beta (3, 6.7)
Fraction of bare skin on hands contacting residues per time	1/min	Beta (9.4, 3.3)
Fraction of bare skin on body (non-hands) contacting residues per time	1/min	Beta (3.1, 16.5)
Fraction of bare skin on hands contacting soil per time	1/min	Beta (9.4, 3.3)
Fraction of bare skin on body (non-hands) contacting soil per time	1/min	Beta (3.1, 16.5)
Daily soil ingestion rate	mg/day	Lognormal (31, 4)
Soil–skin adherence factor	mg/cm^2^	Lognormal (0.11, 2)
Maximum dermal loading for body^a^	μg/cm^2^	Point (0.0105)
Maximum dermal loading for hands^a^	μg/cm^2^	Point (0.0105)
Fraction of hand surface area mouthed per mouthing event		Beta (3.7, 25)
Frequency of hand–mouth activity per hour	Events/hr	Weibull (0.73, 6.93)
Hand washing events per day	Events/day	Lognormal (3.74, 2.63)
Hand washing removal efficiency		Beta (32, 22)
Bathing removal efficiency		Beta (17.1, 5.1)
Typical number of days between baths	Days	Point (1)
Hand–mouth dermal transfer fraction		Beta (14.5, 4.1)
Dermal absorption fraction per day for residues	1/day	Beta (50, 1,611)
Dermal absorption fraction per day for soil	1/day	Beta (50, 1,611)
GI absorption fraction per day for residues	1/day	Beta (4.7, 12.5)
GI absorption fraction per day for soil	1/day	Beta (11.4, 13)

The terms “child” and “children” refer to children 1–6 years of age in the United States who contact CCA-treated wood residues and/or CCA-containing soil from public playsets, at a minimum.

a Input differs from that used by the U.S. EPA (2005), which used a lognormal (0.033, 2.46) distribution (special analyses) or the product of values randomly selected from the residue–skin transfer efficiency and wood surface residue distributions.

**Table 4:** Probabilistic estimates (mean and percentile) of LADD (mg/kg/day × 10^−6^) for children exposed to CCA dislodgeable residues and contaminated soil from treated wood playground structures and residential decks.

	Estimates derived by U.S. EPA (2005)					Estimates derived using the Kwon et al. data				
Pathway	Mean	50th	75th	95th	99th	Mean	50th	75th	95th	99th
Total (playset + deck)	11.0	6.1	13.0	39.0	84.0	3.0	2.7	3.7	5.6	8.1
Total playset	5.4	3.0	5.9	18.0	38.0	1.7	1.4	2.2	3.9	7.0
Residue ingestion from playset	3.1	1.2	3.3	12.0	26.0	0.7	0.6	0.9	1.8	2.5
Soil ingestion from playset	0.7	0.2	0.7	2.8	6.7	0.5	0.2	0.6	2.1	4.5
Residue dermal contact from playset	1.5	0.7	1.8	6.0	13.0	0.4	0.4	0.6	0.8	1.1
Soil dermal contact from playset	0.1	0.1	0.2	0.4	0.8	0.1	0.0	0.1	0.2	0.4
Total deck	5.9	2.8	6.6	21.0	48.0	1.2	1.2	1.6	2.3	2.8
Residue ingestion from deck	3.6	1.5	3.9	13.0	33.0	0.7	0.6	0.9	1.4	2.0
Soil ingestion from deck	0.1	0.0	0.1	0.4	1.3	0.1	0.0	0.1	0.4	0.8
Residue dermal contact from deck	2.2	1.0	2.4	8.0	18.0	0.5	0.5	0.6	0.8	1.0
Soil dermal contact from deck	0.0	0.0	0.0	0.1	0.2	0.0	0.0	0.0	0.1	0.1

**Table 5:** Probabilistic estimates (mean and percentile) of intermediate–term ADD (mg/kg/day × 10^−4^) for children exposed to CCA dislodgeable residues and contaminated soil from treated wood playground structures and residential decks.

	Estimates derived by U.S. EPA (2005)					Estimates derived using the Kwon et al. data				
Pathway	Mean	50th	75th	95th	99th	Mean	50th	75th	95th	99th
Total (playset + deck)	1.3	0.7	1.4	4.5	9.6	0.4	0.3	0.5	0.8	1.4
Total playset	0.6	0.3	0.6	2.6	6.4	0.2	0.2	0.3	0.7	1.2
Residue ingestion from playset	0.4	0.1	0.3	1.7	4.1	0.1	0.1	0.1	0.3	0.4
Soil ingestion from playset	0.1	0.0	0.1	0.3	1.2	0.1	0.0	0.1	0.4	0.9
Residue dermal contact from playset	0.2	0.1	0.2	0.7	1.9	0.1	0.0	0.1	0.1	0.2
Soil dermal contact from playset	0.0	0.0	0.0	0.1	0.2	0.0	0.0	0.0	0.0	0.1
Total deck	0.6	0.3	0.7	2.2	5.9	0.1	0.1	0.2	0.3	0.4
Residue ingestion from deck	0.4	0.2	0.4	1.5	3.7	0.1	0.1	0.1	0.2	0.3
Soil ingestion from deck	0.0	0.0	0.0	0.0	0.1	0.0	0.0	0.0	0.0	0.1
Residue dermal contact from deck	0.2	0.1	0.2	0.8	2.0	0.1	0.0	0.1	0.1	0.2
Soil dermal contact from deck	0.0	0.0	0.0	0.0	0.0	0.0	0.0	0.0	0.0	0.0
